# Supplementary material for: Heterogeneity of the effect of the COVID-19 pandemic on the incidence of Metabolic Syndrome onset at a Japanese campus
Source: PeerJ. 2024 Apr 5;12:e17013. doi: 10.7717/peerj.17013 (PMC11000644; doi:10.7717/peerj.17013)
Supplement: Article S1 [file peerj-12-17013-s001.pdf]

Supplemental Article S1. Diagnostic Criteria for Metabolic Syndrome and Flowchart

Japanese criteria (2005)

Criteria (1) along with the presence of two or more criteria from (2) to (4).

(1) Abdominal circumference*:
  - Male: ≥ 85 cm.
  - Female: ≥ 90 cm.

(2) Dyslipidemia: Triglyceride ≥ 150 mg/dL and/or HDL cholesterol < 40 mg/dL

(3) Blood pressure: Systolic ≥ 130 mmHg and/or diastolic ≥ 85 mmHg

(4) Fasting blood sugar ≥ 110 mg/dL

International criteria (2009)

Presence of any three or more of the following:

(1) Waist circumference*: Population- and country-specific definitions[†]

(2) Triglyceride ≥ 150 mg/dL

(3) HDL cholesterol:
  - Male: <40 mg/dL
  - Female: <50 mg/dL

(4) Blood pressure: Systolic ≥ 130 mmHg and/or diastolic ≥ 85 mmHg

(5) Fasting blood sugar ≥ 100 mg/dL

* Note: The measurement position differs between Japanese and international criteria. The measurement position of international criteria is the midpoint height between the lower margin of the ribs and the superior iliac crest. In contrast, the position of Japanese criteria is the height of the umbilicus. In other words, the Japanese criteria defined the position slightly lower than that of the international criteria.

† Current recommended waist circumference thresholds for Asian (including Japanese) population

- Male: ≥90 cm
- Female: ≥80 cm

Reference:

- An examination committee for criterion of metabolic syndrome. 2005. Definition and criteria of metabolic syndrome. *Nippon Naika Gakkai Zasshi* 94:794-809. (Japanese). DOI: 10.2169/naika.94.794.
- Alberti KGMM, Eckel RH, Grundy SM, Zimmet PZ, Cleeman JI, Donato KA, Fruchart J-C, James WPT, Loria CM, Smith SC Jr. 2009. Harmonizing the metabolic syndrome: a joint interim statement of the International Diabetes Federation Task Force on Epidemiology and Prevention; National Heart, Lung, and

Blood Institute; American Heart Association; World Heart Federation; International Atherosclerosis Society; and International Association for the Study of Obesity. *Circulation* 120:1640-1645. DOI: 10.1161/CIRCULATIONAHA.109.192644.

Flowchart 1

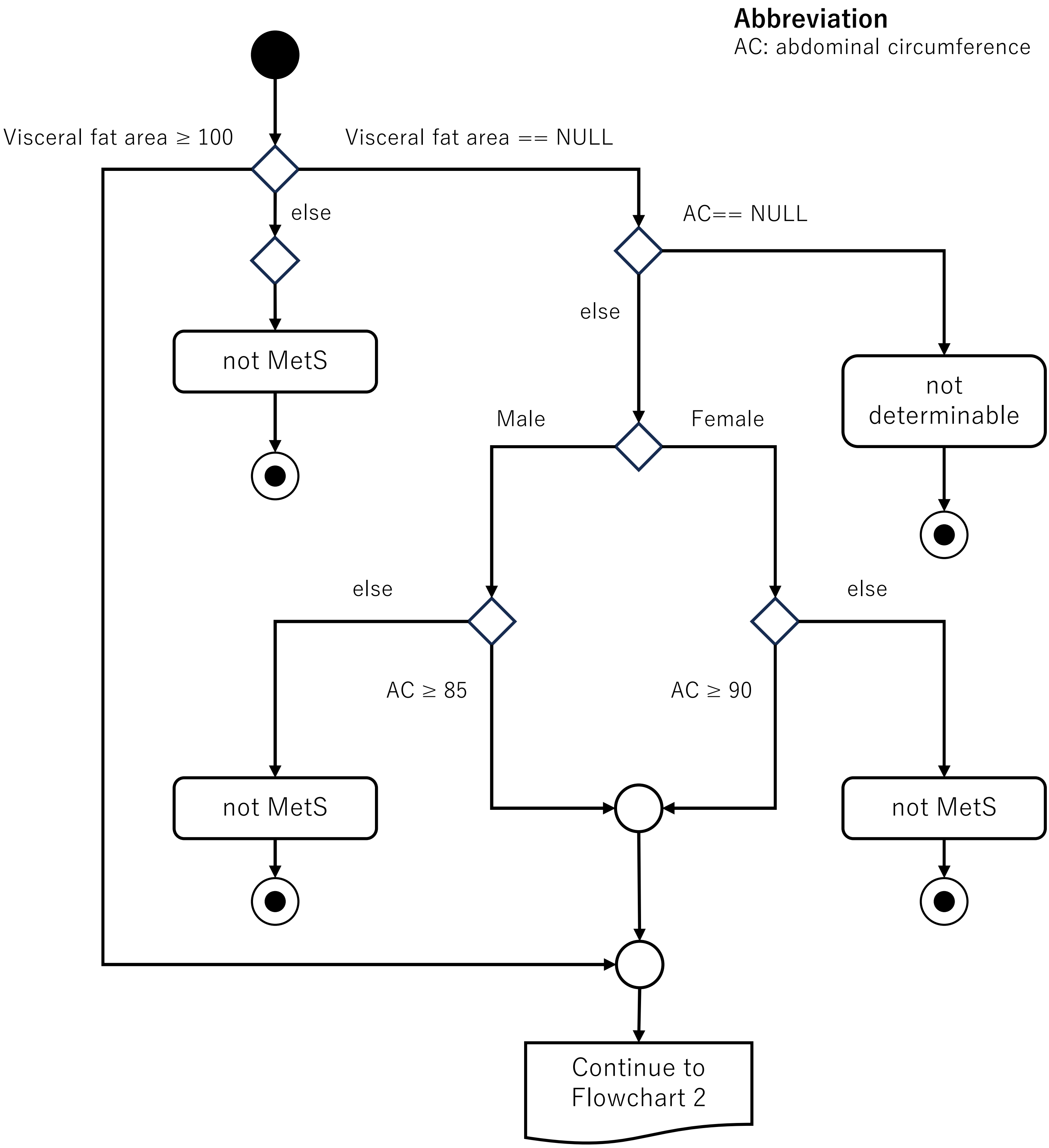

Note:

1. "NULL" includes missing, unmeasured, and unimplemented.
2. If the abdominal circumference is only a self-reported value and the BMI is 22 or higher, the abdominal circumference is considered a missing value.
3. Measurements of abdominal circumference shall be used in the following order of priority for this flowchart:
   ① Medical staff-measured value
   ② Self-measured value
   ③ Self-reported value

Flowchart 2

Abbreviation
FBS: Fasting blood sugar
BS: Blood sugar
HbA1c: Hemoglobin A1c (NGSP)
BS drug: Medication (blood glucose treatment)

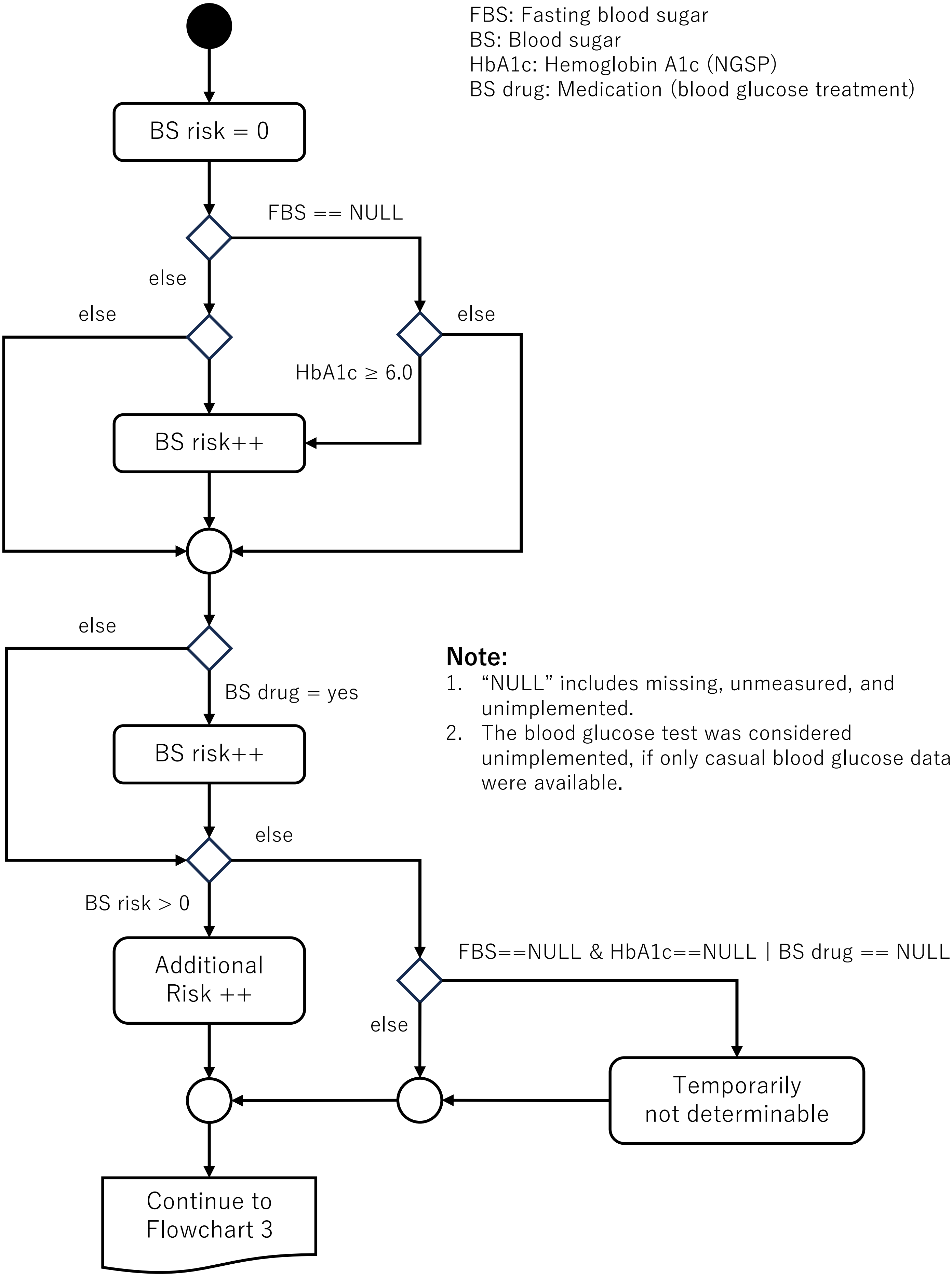

Note:

1. “NULL” includes missing, unmeasured, and unimplemented.
2. The blood glucose test was considered unimplemented, if only casual blood glucose data were available.

Flowchart 3

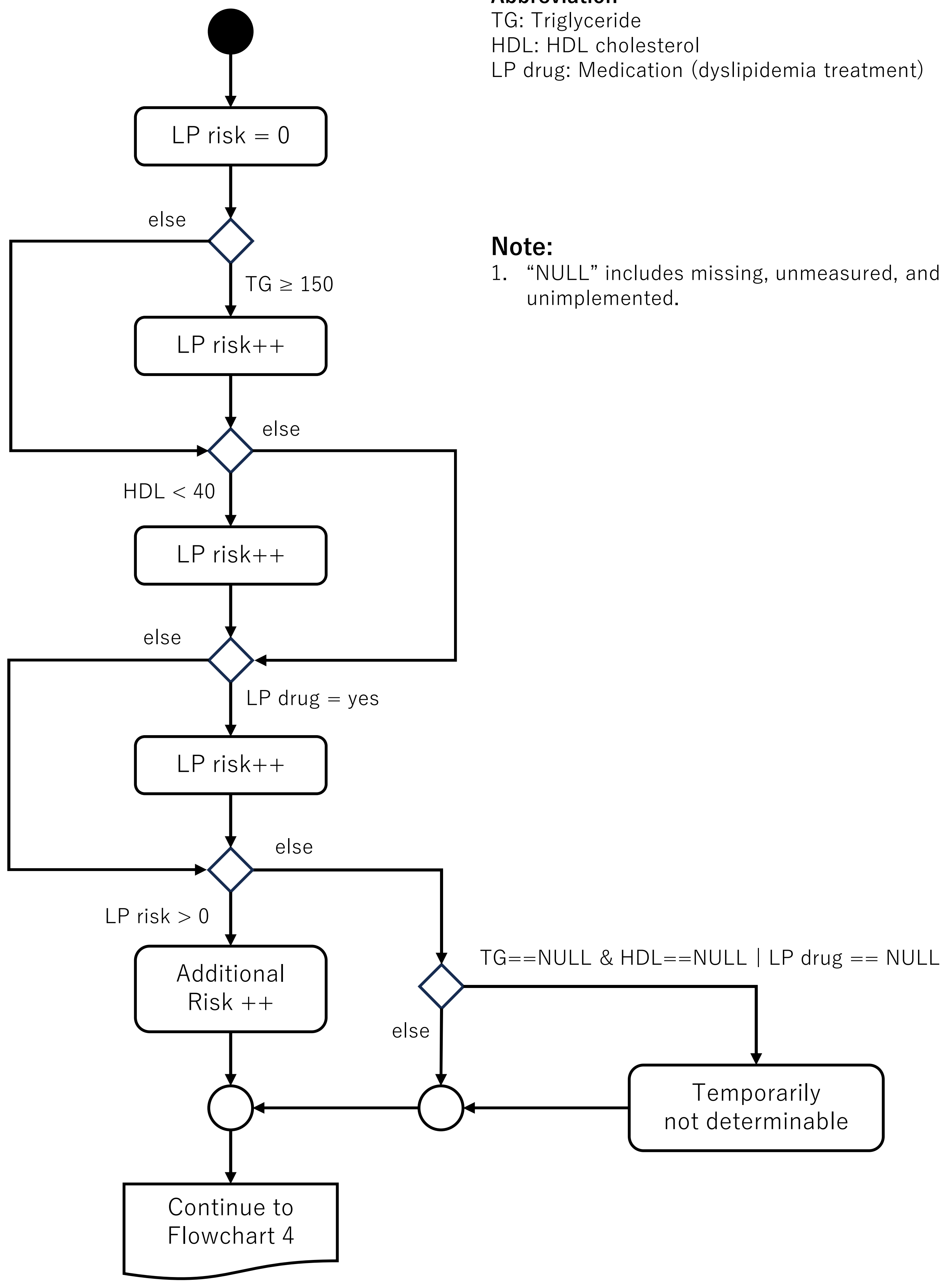

Abbreviation
TG: Triglyceride
HDL: HDL cholesterol
LP drug: Medication (dyslipidemia treatment)

Note:

1. "NULL" includes missing, unmeasured, and unimplemented.

Flowchart 4

Abbreviation
BP: Blood pressure
SBP: Systolic blood pressure
DBP: Diastolic blood pressure
BP drug: Medication (Hypertension treatment)

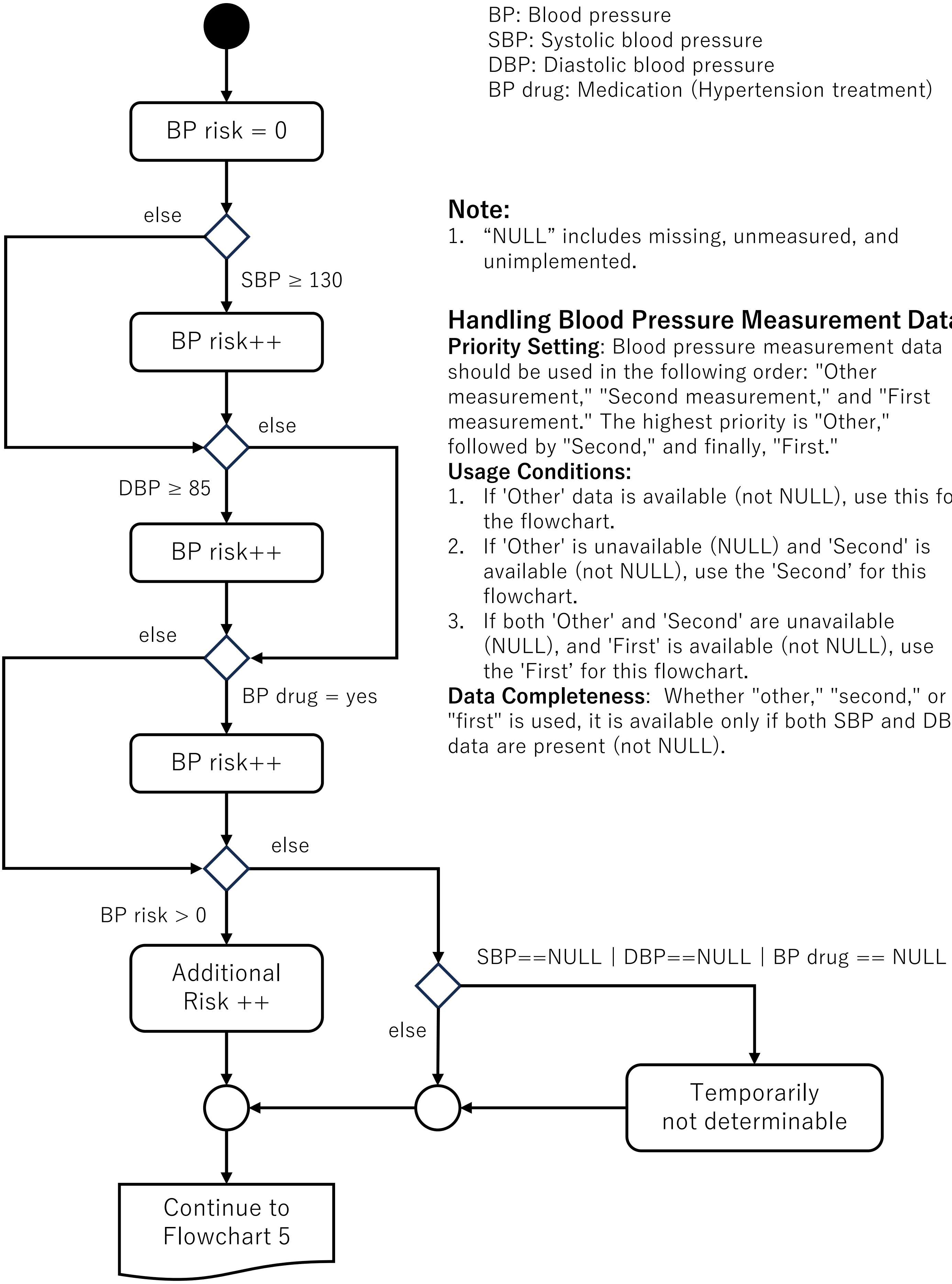

Note:

1. “NULL” includes missing, unmeasured, and unimplemented.

Handling Blood Pressure Measurement Data

Priority Setting: Blood pressure measurement data should be used in the following order: "Other measurement," "Second measurement," and "First measurement." The highest priority is "Other," followed by "Second," and finally, "First."

Usage Conditions:

1. If 'Other' data is available (not NULL), use this for the flowchart.
2. If 'Other' is unavailable (NULL) and 'Second' is available (not NULL), use the 'Second’ for this flowchart.
3. If both 'Other' and 'Second' are unavailable (NULL), and 'First' is available (not NULL), use the 'First’ for this flowchart.

Data Completeness: Whether "other," "second," or "first" is used, it is available only if both SBP and DBP data are present (not NULL).

Flowchart 4

Abbreviation
MetS: Metabolic syndrome
HDL: HDL cholesterol
LP drug: Medication (dyslipidemia treatment)

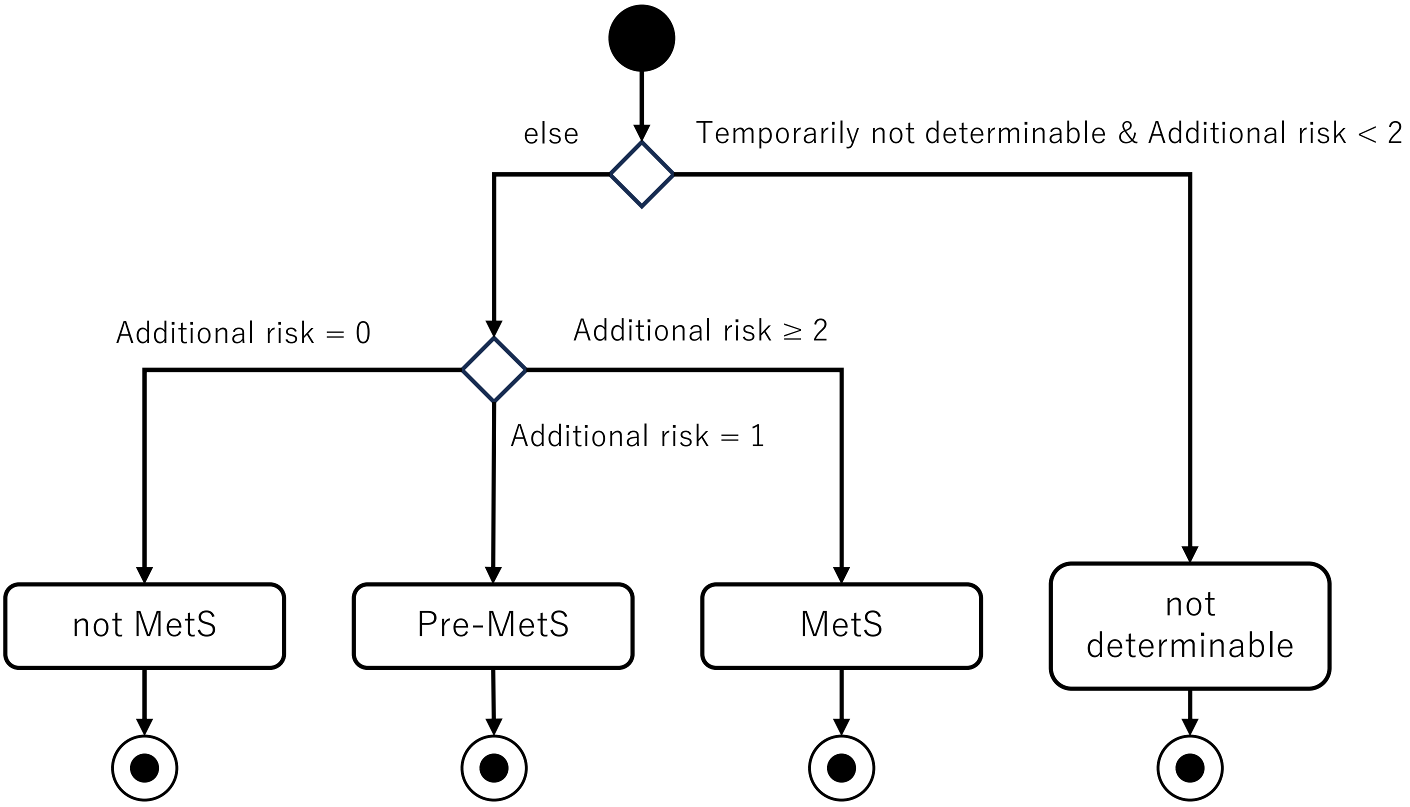

Note:

1. Pre-MetS refers to conditions that are not indicative of metabolic syndrome but are predicted to fall under metabolic syndrome in the near future without appropriate management. This definition is widely used for health guidance in Japan.
