# Supplementary material for: Heterogeneity of the effect of the COVID-19 pandemic on the incidence of Metabolic Syndrome onset at a Japanese campus
Source: PeerJ. 2024 Apr 5;12:e17013. doi: 10.7717/peerj.17013 (PMC11000644; doi:10.7717/peerj.17013)
Supplement: Article S2 [file peerj-12-17013-s002.docx]

# Supplemental Article S2. Items of health checkups.

Japanese law, the ordinance on industrial safety and health, requires that health checkups be conducted at least once a year for all workers. This section lists the items stipulated by that law.

## Excerpt from the Ordinance on Industrial Safety and Health Article 44.

Items of health checkups.

(1) Previous medical history and work history

(2) Subjective symptoms

(3) Height, weight, abdominal circumference, vision, and hearing

(4) Chest X-ray and sputum examination

(5) Blood pressure

(6) Anemia (Hgb and RBC)

(7) Liver function (AST, ALT, γ-GTP)

(8) Blood lipid (LDL/HDL-cholesterol, TG)

(9) Blood glucose test (BS, HbA1c)

(10) Urinalysis (urinary protein, sugar, and occult blood)

(11) Electrocardiogram

Some items can be omitted at the doctor's discretion. Nowadays, in almost all cases, sputum examinations are omitted for workers' health checkups.

## Abbreviations

Hgb, Hemoglobin; RBC, red blood cell; AST, aspartate aminotransferase; ALT, alanine aminotransferase; LDL, low-density lipoprotein; HDL, high-density lipoprotein; TG, triglyceride; HbA1c, hemoglobin A1c; BS, blood Sugar.
