# Supplementary material for: Heterogeneity of the effect of the COVID-19 pandemic on the incidence of Metabolic Syndrome onset at a Japanese campus
Source: PeerJ. 2024 Apr 5;12:e17013. doi: 10.7717/peerj.17013 (PMC11000644; doi:10.7717/peerj.17013)
Supplement: Article S3 [file peerj-12-17013-s003.docx]

# Supplemental Article S3. Constructing Base learners of X-learner

The statistical models and evaluation indices constructed for each base learner M0, M1, M2, and M3 are shown in the table below.

|  | **Constructed by …** | **Evaluated by …** |
| --- | --- | --- |
| Base learner M0 and M1 | Logistic regression model | AUC (Area Under the Curve) |
| Base learner M2 and M3 | Beta regression model | RMSE (Root Mean Squared Error) |

## Base-leaner M0 and M1（Figure 1）

A logistic regression model was used to predict outcomes from predictor variables in the exposed and non-exposed subsamples of the original dataset. The Area Under the Curve (AUC) of the 10-fold cross-validation was the largest for both models that employed the following predictor variables. By the way, the main text of the paper was analyzed using the anonymized dataset.

The predictive variables of M0 and M1

Sex, Division, Age, Age squared, BMI, BMI squared, Abdominal circumference, Abdominal circumference squared, Hearing test 1000Hz/4000Hz right and left, Systolic blood pressure, Systolic blood pressure squared, Diastolic blood pressure, Diastolic blood pressure squared, Urinary protein, Urinary occult blood, AST, ALT, γ-GTP, Fasting blood sugar, Casual blood glucose, HbA1c, HDL cholesterol, HDL cholesterol squared, LDL cholesterol, LDL cholesterol squared, Fasting triglyceride, Casual triglyceride, Hemoglobin, Hemoglobin squared, Red blood cell, ECG finding, Chest X-ray finding, Smoking status, Number of cigarettes, Number of cigarettes squared, Smoking year, History of treatment for hyperlipidemia, History of treatment for hypertension, Missing indicator for casual blood glucose, Missing indicator for fasting blood glucose, Missing indicator for fasting triglyceride, Missing indicator for the number of cigarettes, Missing indicator urinary protein, BMI×Age, Abdominal circumference×Age, AST×ALT, Sex×HbA1c, Number of cigarettes×Smoking year

AUC in the 10-fold cross-validation

|  | M0 | M1 |
| --- | --- | --- |
| Original Dataset | 0.94257 | 0.91226 |
| Anonymized Dataset | 0.95055 | 0.89734 |

## Base-learner M2 and M3 (Figure 1)

A beta regression model was used to predict the Imputed Treatment Effect (ITE) transformed from predictor variables in the exposed/unexposed subsamples of the original dataset. The transformation was performed using the following equation. The original ITE is a real number in the range of (-1, 1), while the transformed ITE' is a real number in the range of (0, 1), which is suitable for the beta regression model.

$$ITE'=\frac{ITE+1}{2}$$

The model employing the following predictors, respectively, minimized the Root Mean Squared Error (RMSE) of the 10-fold cross-validation. The analysis of the main text of the paper was conducted using the anonymized dataset.

The Predictive variables of M2

Sex, Division, Age, Age squared, BMI, BMI squared, Abdominal circumference, Abdominal circumference squared, Hearing test 1000Hz/4000Hz right and left, Systolic blood pressure, Systolic blood pressure squared, Diastolic blood pressure, Diastolic blood pressure squared, Urinary protein, Urinary occult blood, AST, ALT, γ-GTP, Fasting blood sugar, Casual blood glucose, HbA1c, HDL cholesterol, HDL cholesterol squared, LDL cholesterol, LDL cholesterol squared, Fasting triglyceride, Casual triglyceride, Hemoglobin, Hemoglobin squared, Red blood cell, ECG finding, Chest X-ray finding, Smoking status, Number of cigarettes, Number of cigarettes squared, Smoking year, History of treatment for hyperlipidemia, History of treatment for hypertension, Missing indicator for casual blood glucose, Missing indicator for fasting blood glucose, Missing indicator for fasting triglyceride, Missing indicator for the number of cigarettes, Missing indicator urinary protein, BMI×Age, Abdominal circumference×Age, AST×ALT, Sex×Age

The Predictive variables of M3

Sex, Division, Age, Age squared, BMI, BMI squared, Abdominal circumference, Abdominal circumference squared, Hearing test 4000Hz right and left, Systolic blood pressure, Systolic blood pressure squared, Diastolic blood pressure, Diastolic blood pressure squared, Urinary protein, AST, ALT, γ-GTP, Fasting blood sugar, Casual blood glucose, HbA1c, HDL cholesterol, HDL cholesterol squared, LDL cholesterol, LDL cholesterol squared, Fasting triglyceride, Casual triglyceride, Hemoglobin, Hemoglobin squared, Red blood cell, ECG finding, Chest X-ray finding, Smoking status, Number of cigarettes, Number of cigarettes squared, Smoking year, History of treatment for hyperlipidemia, History of treatment for hypertension, Missing indicator for casual blood glucose, Missing indicator for fasting blood glucose, Missing indicator for fasting triglyceride, Missing indicator for the number of cigarettes, Missing indicator urinary protein, BMI×Age, Abdominal circumference×Age, AST×ALT, Sex×Age

RMSE in the 10-fold cross-validation

|  | M2 | M3 |
| --- | --- | --- |
| Original Dataset | 0.08666 | 0.12203 |
| Anonymized Dataset | 0.10096 | 0.14933 |

Sensitivity analysis (robustness assessment for independence assumption)

The pre-COVID-19 data in this study include the same person up to two times (those who took the medical examinations in three consecutive years from 2017 to 2019), which raises concerns about violations of the independence assumption. This section describes the results of our robustness assessment to the base learner.

Note that the anonymized data used in the main analysis cannot identify the same person due to anonymization. Therefore, the original data was used in this validation.

The construction of base learners m0 and m2 used a dataset containing the same individuals (see table below). If a participant has received three health checkups from 2017 to 2019, two of the measurements are nested within that participant's data in the original analysis. The base learner using this data was evaluated as shown in the table above, with AUC = 0.94257 for m0 and RMSE = 0.08666 for m2.

Differences in analytical targets in ex-ante analysis and sensitivity analysis

| Data for analysis | Baseline | Follow up |
| --- | --- | --- |
| Original analysis | Health checkup in…  FY 2017  FY 2018 | Health checkup in…  FY 2018  FY 2019 |
| Sensitivity analysis | Health checkup in…  FY 2017 | Health checkup in…  FY 2018 |

I then analyze the same using only a subset of the unduplicated data (2017 covariates and 2018 outcomes). The result was an AUC=0.93782 for base learner m0 and a rating of RMSE=0.10604 for base learner m2.

This raised concerns that the model may have been overly accurate due to duplicate data for the same person, but the difference was insignificant. So, it was judged to be within acceptable limits for this study's results.

Since base learners m1 and m3 do not have duplicate data for the same person, this sensitivity analysis was not performed.

Comparison of base-learner performance in the original analysis and sensitivity analysis

|  | M0 | M2 |
| --- | --- | --- |
| Original analysis | 0.94257 | 0.08666 |
| Sensitivity analysis | 0.93782 | 0.10604 |
