# Supplementary material for: Heterogeneity of the effect of the COVID-19 pandemic on the incidence of Metabolic Syndrome onset at a Japanese campus
Source: PeerJ. 2024 Apr 5;12:e17013. doi: 10.7717/peerj.17013 (PMC11000644; doi:10.7717/peerj.17013)
Supplement: Table S1 — CATE, Conditional Average Treatment Effect, CI, Confident Interval. 95%CI is calculated by the bootstrap method. [file peerj-12-17013-s004.docx]

Supplemental Table S1. CATEs and their 95% CI for overall, by division, and by gender (original dataset)

|  | Number | CATE | 95%CI | | p-value |
| --- | --- | --- | --- | --- | --- |
| Total | 3,752 | 0.036 | -0.000 | 0.072 | 0.053 |
| Administrative division | 837 | 0.049 | -0.009 | 0.106 | 0.096 |
| Research division | 435 | 0.069 | -0.018 | 0.156 | 0.121 |
| Medical division | 2,101 | 0.016 | -0.024 | 0.057 | 0.428 |
| Intensive Care division | 199 | 0.116 | -0.024 | 0.256 | 0.104 |
| Female | 2,444 | 0.022 | -0.006 | 0.050 | 0.119 |
| Male | 1,128 | 0.066 | -0.014 | 0.146 | 0.105 |

CATE: Conditional Average Treatment Effect, CI: Confident Interval.

95%CI is calculated by the bootstrap method.
