# Supplementary material for: Heterogeneity of the effect of the COVID-19 pandemic on the incidence of Metabolic Syndrome onset at a Japanese campus
Source: PeerJ. 2024 Apr 5;12:e17013. doi: 10.7717/peerj.17013 (PMC11000644; doi:10.7717/peerj.17013)
Supplement: Table S2 [file peerj-12-17013-s005.docx]

Supplemental Table S2. Differences in CATE between categories and their 95% confidence intervals according to division and sex (original dataset).

|  | Number | Difference in CATE | 95% CI |  | p-value |
| --- | --- | --- | --- | --- | --- |
| Administrative division | 837 | 0.032 | -0.031 | 0.095 | 0.320 |
| Research division | 435 | 0.052 | -0.035 | 0.140 | 0.238 |
| Medical division | 2,101 | 0 | (reference category) | | |
| Intensive Care division | 199 | 0.100 | -0.039 | 0.238 | 0.158 |
| Female sex | 2,410 | 0 | (reference category) | | |
| Male sex | 1,162 | 0.044 | -0.030 | 0.119 | 0.249 |

CATE, Conditional Average Treatment Effect; CI, Confident Interval.

95% CI was calculated by the bootstrap method.

In division, medical division was used as the reference category, and in sex, female sex was used as the reference category.
