# Supplementary figures and images for: Heterogeneity of the effect of the COVID-19 pandemic on the incidence of Metabolic Syndrome onset at a Japanese campus
Source: PeerJ. 2024 Apr 5;12:e17013. doi: 10.7717/peerj.17013 (PMC11000644; doi:10.7717/peerj.17013)

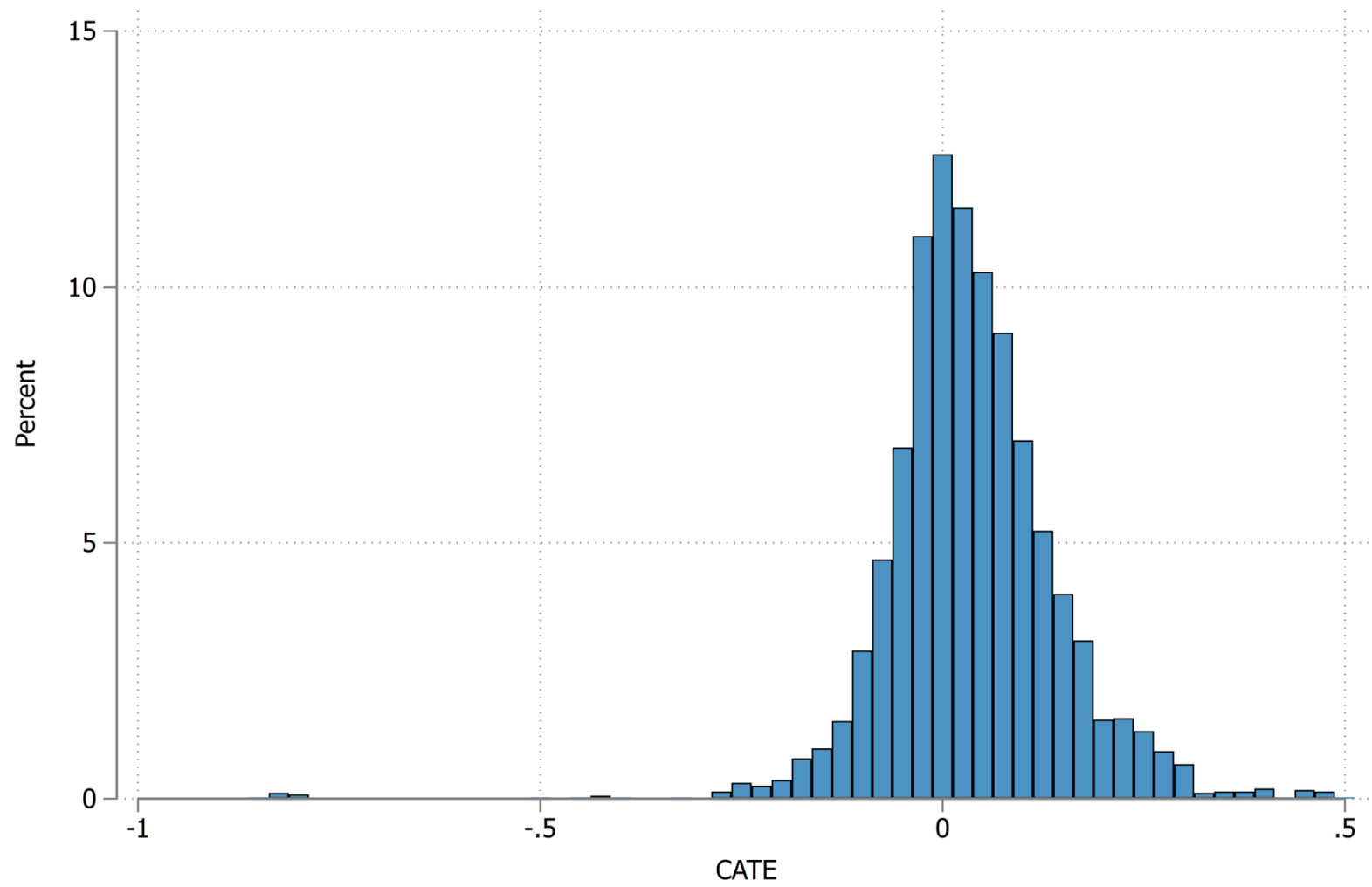

Supplement: Figure S1 [file peerj-12-17013-s006.pdf]

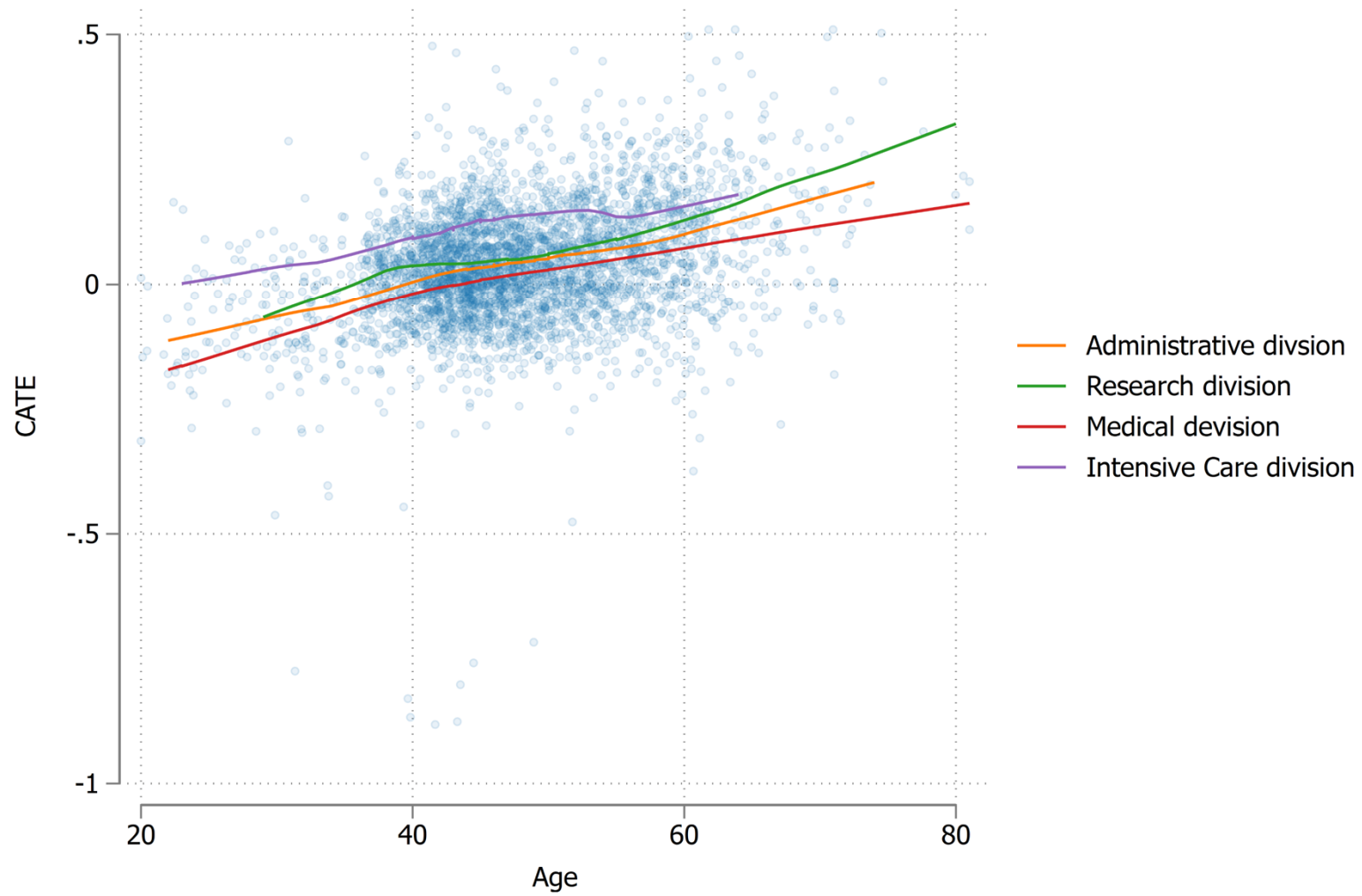

Supplement: Figure S2 [file peerj-12-17013-s007.pdf]
